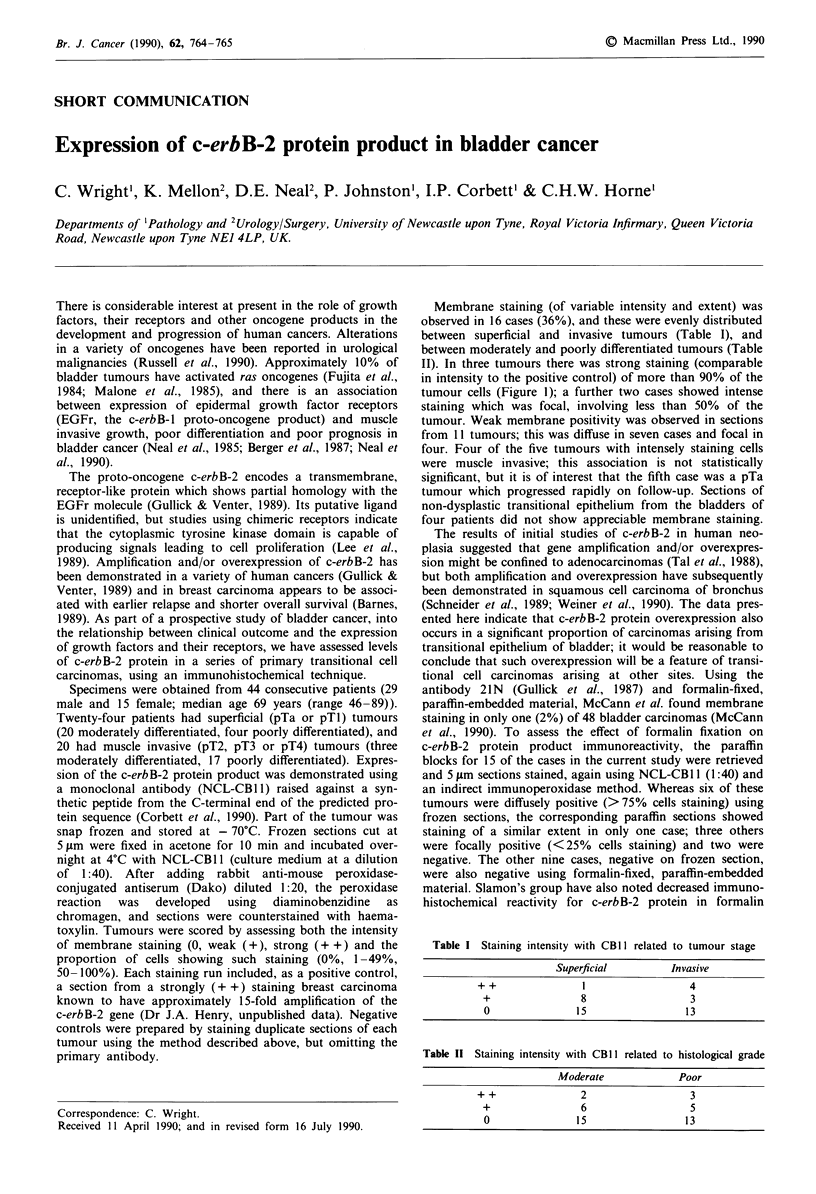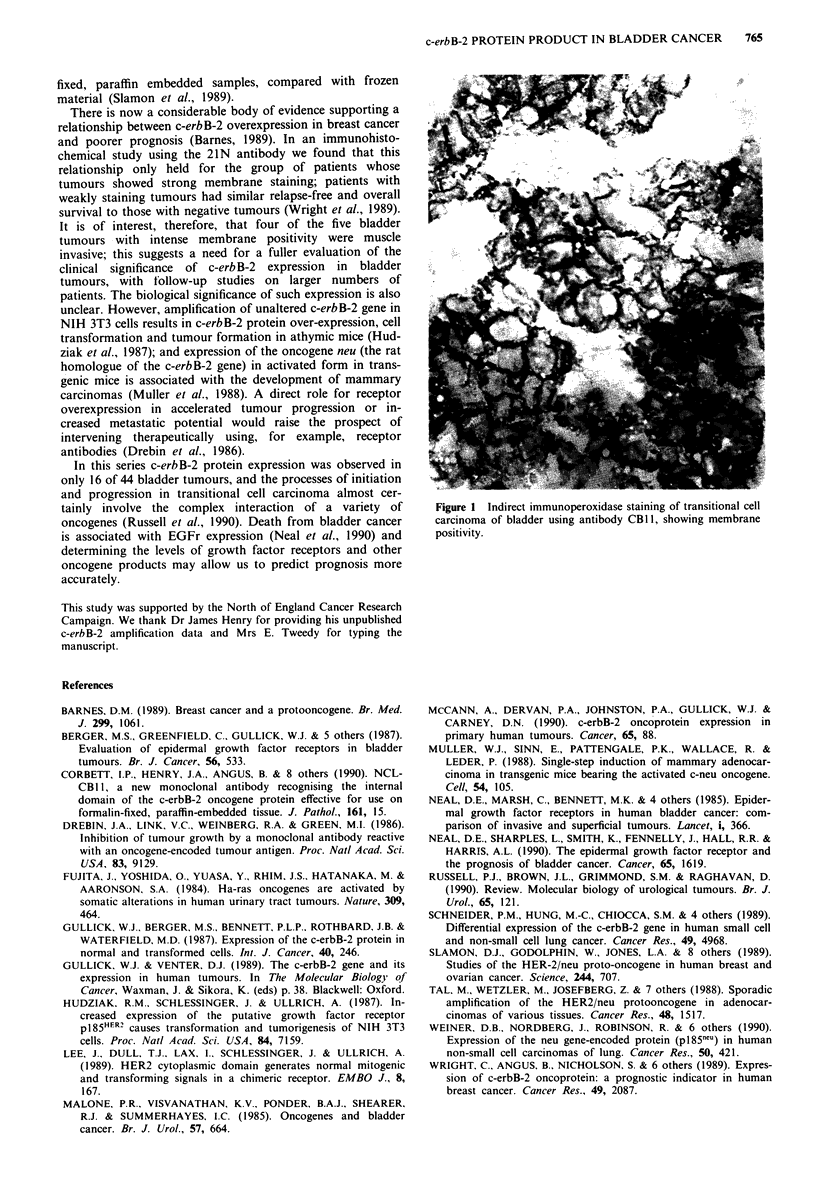# Expression of c-erbB-2 protein product in bladder cancer.

**DOI:** 10.1038/bjc.1990.375

**Published:** 1990-11

**Authors:** C. Wright, K. Mellon, D. E. Neal, P. Johnston, I. P. Corbett, C. H. Horne

**Affiliations:** Department of Pathology, University of Newcastle upon Tyne, Royal Victoria Infirmary, UK.

## Abstract

**Images:**


SHORT COMMUNICATION

### Expression of c-*erb*B-2 protein product in bladder cancer

C. Wright[1], K. Mellon[2], D.E. Neal[2], P. Johnston[1], I.P. Corbett[1] & C.H.W. Horne[1]

*Departments of [1]Pathology and [2]Urology/Surgery, University of Newcastle upon Tyne, Royal Victoria Infirmary, Queen Victoria Road, Newcastle upon Tyne NE1 4LP, UK.*

There is considerable interest at present in the role of growth factors, their receptors and other oncogene products in the development and progression of human cancers. Alterations in a variety of oncogenes have been reported in urological malignancies (Russell *et al.*, 1990). Approximately 10% of bladder tumours have activated *ras* oncogenes (Fujita *et al.*, 1984; Malone *et al.*, 1985), and there is an association between expression of epidermal growth factor receptors (EGFr, the c-*erb*B-1 proto-oncogene product) and muscle invasive growth, poor differentiation and poor prognosis in bladder cancer (Neal *et al.*, 1985; Berger *et al.*, 1987; Neal *et al.*, 1990).

The proto-oncogene c-*erb*B-2 encodes a transmembrane, receptor-like protein which shows partial homology with the EGFr molecule (Gullick & Venter, 1989). Its putative ligand is unidentified, but studies using chimeric receptors indicate that the cytoplasmic tyrosine kinase domain is capable of producing signals leading to cell proliferation (Lee *et al.*, 1989). Amplification and/or overexpression of c-*erb*B-2 has been demonstrated in a variety of human cancers (Gullick & Venter, 1989) and in breast carcinoma appears to be associated with earlier relapse and shorter overall survival (Barnes, 1989). As part of a prospective study of bladder cancer, into the relationship between clinical outcome and the expression of growth factors and their receptors, we have assessed levels of c-*erb*B-2 protein in a series of primary transitional cell carcinomas, using an immunohistochemical technique.

Specimens were obtained from 44 consecutive patients (29 male and 15 female; median age 69 years (range 46-89)). Twenty-four patients had superficial (pTa or pT1) tumours (20 moderately differentiated, four poorly differentiated), and 20 had muscle invasive (pT2, pT3 or pT4) tumours (three moderately differentiated, 17 poorly differentiated). Expression of the c-*erb*B-2 protein product was demonstrated using a monoclonal antibody (NCL-CB11) raised against a synthetic peptide from the C-terminal end of the predicted protein sequence (Corbett *et al.*, 1990). Part of the tumour was snap frozen and stored at − 70°C. Frozen sections cut at 5 μm were fixed in acetone for 10 min and incubated overnight at 4°C with NCL-CB11 (culture medium at a dilution of 1:40). After adding rabbit anti-mouse peroxidase-conjugated antiserum (Dako) diluted 1:20, the peroxidase reaction was developed using diaminobenzidine as chromagen, and sections were counterstained with haematoxylin. Tumours were scored by assessing both the intensity of membrane staining (0, weak (+), strong (+ +) and the proportion of cells showing such staining (0%, 1-49%, 50-100%). Each staining run included, as a positive control, a section from a strongly (+ +) staining breast carcinoma known to have approximately 15-fold amplification of the c-*erb*B-2 gene (Dr J.A. Henry, unpublished data). Negative controls were prepared by staining duplicate sections of each tumour using the method described above, but omitting the primary antibody.

Membrane staining (of variable intensity and extent) was observed in 16 cases (36%), and these were evenly distributed between superficial and invasive tumours (Table I), and between moderately and poorly differentiated tumours (Table II). In three tumours there was strong staining (comparable in intensity to the positive control) of more than 90% of the tumour cells (Figure 1); a further two cases showed intense staining which was focal, involving less than 50% of the tumour. Weak membrane positivity was observed in sections from 11 tumours; this was diffuse in seven cases and focal in four. Four of the five tumours with intensely staining cells were muscle invasive; this association is not statistically significant, but it is of interest that the fifth case was a pTa tumour which progressed rapidly on follow-up. Sections of non-dysplastic transitional epithelium from the bladders of four patients did not show appreciable membrane staining.

The results of initial studies of c-*erb*B-2 in human neoplasia suggested that gene amplification and/or overexpression might be confined to adenocarcinomas (Tal *et al.*, 1988), but both amplification and overexpression have subsequently been demonstrated in squamous cell carcinoma of bronchus (Schneider *et al.*, 1989; Weiner *et al.*, 1990). The data presented here indicate that c-*erb*B-2 protein overexpression also occurs in a significant proportion of carcinomas arising from transitional epithelium of bladder; it would be reasonable to conclude that such overexpression will be a feature of transitional cell carcinomas arising at other sites. Using the antibody 21N (Gullick *et al.*, 1987) and formalin-fixed, paraffin-embedded material, McCann *et al.* found membrane staining in only one (2%) of 48 bladder carcinomas (McCann *et al.*, 1990). To assess the effect of formalin fixation on c-*erb*B-2 protein product immunoreactivity, the paraffin blocks for 15 of the cases in the current study were retrieved and 5 μm sections stained, again using NCL-CB11 (1:40) and an indirect immunoperoxidase method. Whereas six of these tumours were diffusely positive (>75% cells staining) using frozen sections, the corresponding paraffin sections showed staining of a similar extent in only one case; three others were focally positive (<25% cells staining) and two were negative. The other nine cases, negative on frozen section, were also negative using formalin-fixed, paraffin-embedded material. Slamon's group have also noted decreased immunohistochemical reactivity for c-*erb*B-2 protein in formalin

**Table I** Staining intensity with CB11 related to tumour stage

| | *Superficial* | *Invasive* |
|---|---|---|
| + + | 1 | 4 |
| + | 8 | 3 |
| 0 | 15 | 13 |

**Table II** Staining intensity with CB11 related to histological grade

| | *Moderate* | *Poor* |
|---|---|---|
| + + | 2 | 3 |
| + | 6 | 5 |
| 0 | 15 | 13 |

Correspondence: C. Wright.
Received 11 April 1990; and in revised form 16 July 1990.

fixed, paraffin embedded samples, compared with frozen material (Slamon *et al.*, 1989).

There is now a considerable body of evidence supporting a relationship between c-*erb*B-2 overexpression in breast cancer and poorer prognosis (Barnes, 1989). In an immunohistochemical study using the 21N antibody we found that this relationship only held for the group of patients whose tumours showed strong membrane staining; patients with weakly staining tumours had similar relapse-free and overall survival to those with negative tumours (Wright *et al.*, 1989). It is of interest, therefore, that four of the five bladder tumours with intense membrane positivity were muscle invasive; this suggests a need for a fuller evaluation of the clinical significance of c-*erb*B-2 expression in bladder tumours, with follow-up studies on larger numbers of patients. The biological significance of such expression is also unclear. However, amplification of unaltered c-*erb*B-2 gene in NIH 3T3 cells results in c-*erb*B-2 protein over-expression, cell transformation and tumour formation in athymic mice (Hudziak *et al.*, 1987); and expression of the oncogene *neu* (the rat homologue of the c-*erb*B-2 gene) in activated form in transgenic mice is associated with the development of mammary carcinomas (Muller *et al.*, 1988). A direct role for receptor overexpression in accelerated tumour progression or increased metastatic potential would raise the prospect of intervening therapeutically using, for example, receptor antibodies (Drebin *et al.*, 1986).

In this series c-*erb*B-2 protein expression was observed in only 16 of 44 bladder tumours, and the processes of initiation and progression in transitional cell carcinoma almost certainly involve the complex interaction of a variety of oncogenes (Russell *et al.*, 1990). Death from bladder cancer is associated with EGFr expression (Neal *et al.*, 1990) and determining the levels of growth factor receptors and other oncogene products may allow us to predict prognosis more accurately.

**Figure 1** Indirect immunoperoxidase staining of transitional cell carcinoma of bladder using antibody CB11, showing membrane positivity.

This study was supported by the North of England Cancer Research Campaign. We thank Dr James Henry for providing his unpublished c-*erb*B-2 amplification data and Mrs E. Tweedy for typing the manuscript.

### References

BARNES, D.M. (1989). Breast cancer and a protooncogene. *Br. Med. J.* **299**, 1061.

BERGER, M.S., GREENFIELD, C., GULLICK, W.J. & 5 others (1987). Evaluation of epidermal growth factor receptors in bladder tumours. *Br. J. Cancer*, **56**, 533.

CORBETT, I.P., HENRY, J.A., ANGUS, B. & 8 others (1990). NCL-CB11, a new monoclonal antibody recognising the internal domain of the c-erbB-2 oncogene protein effective for use on formalin-fixed, paraffin-embedded tissue. *J. Pathol.*, **161**, 15.

DREBIN, J.A., LINK, V.C., WEINBERG, R.A. & GREEN, M.I. (1986). Inhibition of tumour growth by a monoclonal antibody reactive with an oncogene-encoded tumour antigen. *Proc. Natl Acad. Sci. USA*, **83**, 9129.

FUJITA, J., YOSHIDA, O., YUASA, Y., RHIM, J.S., HATANAKA, M. & AARONSON, S.A. (1984). Ha-ras oncogenes are activated by somatic alterations in human urinary tract tumours. *Nature*, **309**, 464.

GULLICK, W.J., BERGER, M.S., BENNETT, P.L.P., ROTHBARD, J.B. & WATERFIELD, M.D. (1987). Expression of the c-erbB-2 protein in normal and transformed cells. *Int. J. Cancer*, **40**, 246.

GULLICK, W.J. & VENTER, D.J. (1989). The c-erbB-2 gene and its expression in human tumours. In *The Molecular Biology of Cancer*, Waxman, J. & Sikora, K. (eds) p. 38. Blackwell: Oxford.

HUDZIAK, R.M., SCHLESSINGER, J. & ULLRICH, A. (1987). Increased expression of the putative growth factor receptor $p185^{HER2}$ causes transformation and tumorigenesis of NIH 3T3 cells. *Proc. Natl Acad. Sci. USA*, **84**, 7159.

LEE, J., DULL, T.J., LAX, I., SCHLESSINGER, J. & ULLRICH, A. (1989). HER2 cytoplasmic domain generates normal mitogenic and transforming signals in a chimeric receptor. *EMBO J.*, **8**, 167.

MALONE, P.R., VISVANATHAN, K.V., PONDER, B.A.J., SHEARER, R.J. & SUMMERHAYES, I.C. (1985). Oncogenes and bladder cancer. *Br. J. Urol.*, **57**, 664.

McCANN, A., DERVAN, P.A., JOHNSTON, P.A., GULLICK, W.J. & CARNEY, D.N. (1990). c-erbB-2 oncoprotein expression in primary human tumours. *Cancer*, **65**, 88.

MULLER, W.J., SINN, E., PATTENGALE, P.K., WALLACE, R. & LEDER, P. (1988). Single-step induction of mammary adenocarcinoma in transgenic mice bearing the activated c-neu oncogene. *Cell*, **54**, 105.

NEAL, D.E., MARSH, C., BENNETT, M.K. & 4 others (1985). Epidermal growth factor receptors in human bladder cancer: comparison of invasive and superficial tumours. *Lancet*, **i**, 366.

NEAL, D.E., SHARPLES, L., SMITH, K., FENNELLY, J., HALL, R.R. & HARRIS, A.L. (1990). The epidermal growth factor receptor and the prognosis of bladder cancer. *Cancer*, **65**, 1619.

RUSSELL, P.J., BROWN, J.L., GRIMMOND, S.M. & RAGHAVAN, D. (1990). Review. Molecular biology of urological tumours. *Br. J. Urol.*, **65**, 121.

SCHNEIDER, P.M., HUNG, M.-C., CHIOCCA, S.M. & 4 others (1989). Differential expression of the c-erbB-2 gene in human small cell and non-small cell lung cancer. *Cancer Res.*, **49**, 4968.

SLAMON, D.J., GODOLPHIN, W., JONES, L.A. & 8 others (1989). Studies of the HER-2/neu proto-oncogene in human breast and ovarian cancer. *Science*, **244**, 707.

TAL, M., WETZLER, M., JOSEFBERG, Z. & 7 others (1988). Sporadic amplification of the HER2/neu protooncogene in adenocarcinomas of various tissues. *Cancer Res.*, **48**, 1517.

WEINER, D.B., NORDBERG, J., ROBINSON, R. & 6 others (1990). Expression of the neu gene-encoded protein ($p185^{neu}$) in human non-small cell carcinomas of lung. *Cancer Res.*, **50**, 421.

WRIGHT, C., ANGUS, B., NICHOLSON, S. & 6 others (1989). Expression of c-erbB-2 oncoprotein: a prognostic indicator in human breast cancer. *Cancer Res.*, **49**, 2087.